# Transient Reflexive Pain Responses and Chronic Affective Nonreflexive Pain Responses Associated with Neuroinflammation Processes in Both Spinal and Supraspinal Structures in Spinal Cord-Injured Female Mice

**DOI:** 10.3390/ijms24021761

**Published:** 2023-01-16

**Authors:** Sílvia Castany, Anna Bagó-Mas, José Miguel Vela, Enrique Verdú, Karolina Bretová, Viktorie Svobodová, Petr Dubový, Pere Boadas-Vaello

**Affiliations:** 1 Research Group of Clinical Anatomy, Embryology and Neuroscience (NEOMA), Department of Medical Sciences, University of Girona, 17003 Girona, Catalonia, Spain; 2 Department of Anatomy, Division of Neuroanatomy, Faculty of Medicine, Masaryk University, 625 00 Brno, Czech Republic; 3 WeLab Barcelona, Parc Científic de Barcelona, 08028 Barcelona, Catalonia, Spain

**Keywords:** spinal cord injury, central neuropathic pain, chronic pain, neuroinflammation, cytokines, chemokines, gliosis, CX3CL1/CX3CR1

## Abstract

Central neuropathic pain is not only characterized by reflexive pain responses, but also emotional or affective nonreflexive pain responses, especially in women. Some pieces of evidence suggest that the activation of the neuroimmune system may be contributing to the manifestation of mood disorders in patients with chronic pain conditions, but the mechanisms that contribute to the development and chronicity of CNP and its associated disorders remain poorly understood. This study aimed to determine whether neuroinflammatory factor over-expression in the spinal cord and supraspinal structures may be associated with reflexive and nonreflexive pain response development from acute SCI phase to 12 weeks post-injury in female mice. The results show that transient reflexive responses were observed during the SCI acute phase associated with transient cytokine overexpression in the spinal cord. In contrast, increased nonreflexive pain responses were observed in the chronic phase associated with cytokine overexpression in supraspinal structures, especially in mPFC. In addition, results revealed that besides cytokines, the mPFC showed an increased glial activation as well as CX3CL1/CX3CR1 upregulation in the neurons, suggesting the contribution of neuron-glia crosstalk in the development of nonreflexive pain responses in the chronic spinal cord injury phase.

## 1. Introduction

Central neuropathic pain (CNP) is a severe sensory deficit experienced by more than 50% of people with spinal cord injury (SCI) [1,2] and 40–60% of them develop chronic pain [3]. CNP is largely refractory to current pharmacological treatment approaches, and it has a poor prognosis for recovery [2], leading thus to the present scenario where the majority of SCI patients are in the chronic phase [4]. It is worth to note that neuropathic pain is not only characterized by reflexive pain responses (e.g., thermal hyperalgesia and mechanical allodynia) but it is also associated with emotional or affective disturbances, called nonreflexive pain responses, which may be related to the dysfunction of supraspinal structures playing critical roles in these behavioral components. It is known that psychological issues have tremendous importance in the experience and expression of any pain condition [5]. Physical and sensory dysfunction in the form of pain after SCI has a significant impact on the quality of life and psychological well-being of these patients. Indeed, the quality of life in patients with neuropathic pain due to a traumatic SCI is severely compromised [6], and they are also often diagnosed with mood disorders [7,8,9]. This health concern is especially important in females. Epidemiological data highlight a higher prevalence of chronic pain in females and also a higher vulnerability in the development of comorbid pain and emotional disorders [10,11]. It is important to highlight the close reciprocal relationship that occurs between chronic pain and depression [12], probably reflecting common mechanisms and neural pathways between these pathologies.

Some pieces of evidence suggest that the activation of the neuroimmune system may be contributing to the manifestation of mood disorders in patients with chronic pain conditions. Clinical studies have shown an association between elevated levels of proinflammatory cytokines and mood disorders [13,14]. Higher levels of pro-inflammatory cytokines (e.g., tumor necrosis factor TNFα, interleukin IL6 and IL1β) in the central nervous system (CNS) have been suggested to contribute to the pathophysiology of neuropathic pain and the related mood disorder comorbidities [13]. Central or systemic administration of proinflammatory cytokines in rodent models induces sickness behavior characterized by behavioral and physiological changes resembling depression [15] and can be alleviated with antidepressants [16]. These findings suggest that immune system activation in supraspinal regions may play a pivotal role in the development of mood disorders. Indeed, SCI could be considered a neuroimmune disorder since data indicate a critical involvement of innate and adaptive immune responses following injury [17].

However, the mechanisms that contribute to the development and chronicity of CNP and its associated mood disorders remain poorly understood. Such knowledge must urgently be described in order to design new therapeutic strategies aimed at improving the quality of life of SCI patients. Nowadays, well-characterized preclinical models of SCI-induced neuropathic pain are needed since only few models are available for studies up to the SCI-chronic phase [18,19,20,21,22,23,24,25,26]. Moreover, all of them show outcomes below 8 weeks post-injury and are usually accompanied with locomotor disturbances that may disturb pain responses evaluation.

In this context, this study was designed to develop an appropriate translational female mice model of central neuropathic pain after mild spinal cord injury, without major locomotor disturbances, up to 12 weeks post-injury, to study the development of chronic reflexive and nonreflexive pain responses. The nonreflexive responses (e.g., affective measurements) associated with pain processes provide complementary information about the impact of the sensory abnormalities in the function of supraspinal structures [27]. Among the supraspinal structures that play critical roles in affective and emotional behaviors are the nucleus accumbens (NAcc), the medial prefrontal cortex (mPFC) and its anterior cingulate cortex (ACC), as well as the amygdala. The NAcc displays connections with multiple brain regions including the ventral tegmental area, amygdala, thalamus, and mPFC. Optogenetic activation of mPFC-NAcc projections modulates sensory and affective symptoms of neuropathic pain [28] and induces resilience to chronic social defeat stress [29]. On the other hand, several studies have reported plasticity-related alterations in the mPFC of rodent models of neuropathic pain, with both chronic stress and nerve injury resulting in altered dendritic spine morphology [30,31]. Some neuroimaging studies from chronic pain patients show altered activity in the mPFC and NAcc [32,33]. These two brain regions are part of the brain reward center and have documented roles in the pathophysiology of depression [34]. Thus, this study was aimed at determining whether inflammatory cytokine over-expression in the spinal cord and supraspinal structures (mPFC, NAcc, amygdala) may be associated with reflexive and nonreflexive pain response development from the acute SCI-phase to 12 weeks post-injury in female mice. In addition, to gain mechanistic insights related to the results obtained, a final experiment was performed to elucidate whether gliosis and CX3CL1/CX3CR1 expression in the anterior cingulate cortex (ACC) compartments were involved in developing affective nonreflexive pain responses.

## 2. Results

### 2.1. Mild Spinal Cord Injury Results in Chronic Neuropathic Pain without Major Locomotor Disturbances

**Figure 1:**
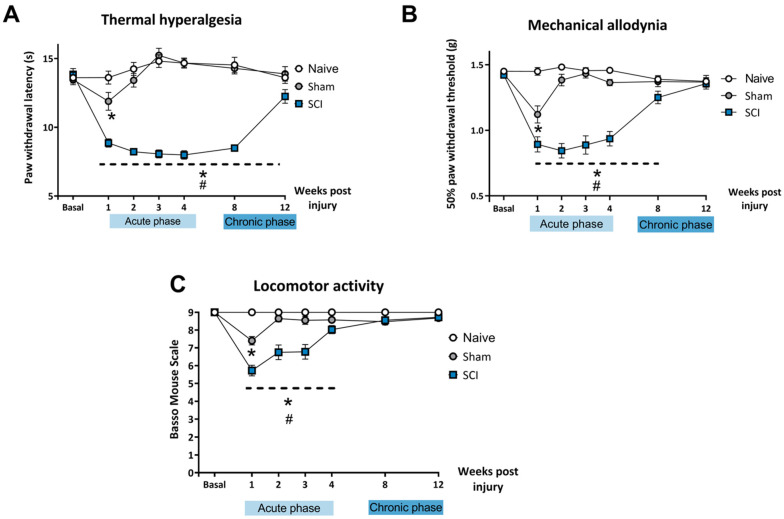
Assessment of mechanical allodynia, thermal hyperalgesia and locomotor activity after a mild spinal cord injury (SCI). Each point and vertical line represent the mean ± SEM. Experimental groups: Naive (n = 18), Sham (n = 28) and SCI (n = 30). (**A**) Thermal hyperalgesia was significantly evidenced in SCI mice until 12 wpi (**B**) Mechanical allodynia was significantly present in SCI mice up to 8 wpi (**C**) Mild BMS alterations referring to altered paw position but not to altered horizontal locomotion were evidenced until 4 wpi. SCI, spinal cord injury; wpi, weeks post injury; BMS, Basso Mouse Scale. * *p* < 0.05 vs. naïve group, # *p* < 0.05 vs. sham (Two-way ANOVA followed by Tukey’s post-hoc).

After mild SCI, mice developed thermal hyperalgesia during the acute phase (1, 2, 3, 4 wpi) and chronic phase (8 wpi, 12 wpi) of the lesion (Figure 1A). Two-way ANOVA analysis indicated significant effects of time (*p* < 0.01) and surgery (*p* < 0.01) factors and significant interaction for time × injury (*p* < 0.01). Post-hoc analysis showed that SCI mice experienced a decrease in paw withdrawal latency in all the evaluated time points when compared to naïve and control mice (*p* < 0.05). The sham experimental group showed significant differences in withdrawal latency time only during the first week after surgery when compared to the naïve group. After 1 wpi, no significant differences were found between naïve and sham control groups.

Similarly, SCI mice also developed mechanical allodynia in the acute phase (1, 2, 3, 4 wpi) and the chronic phase (8 wpi) of the injury, recovering normal mechanical threshold sensitivity at 12 wpi (Figure 1B).

Two-way ANOVA analysis indicated significant effects of time (*p* < 0.01) and surgery (*p* < 0.01) factors and significant interaction for time × injury (*p* < 0.01). Post-hoc analysis showed that mild SCI induced a significant decrease in the paw withdrawal threshold until 8 wpi when compared to naïve and control mice (*p* < 0.05). The sham control group only showed a decrease in withdrawal threshold the first week after surgery when compared to the naïve group. After 1 wpi, no significant differences were found between naïve and sham groups.

Locomotor performance was assessed with BMS, which evaluates the gait pattern and the overall locomotion of the animals. Spinal cord-injured mice experienced a transient impairment of the locomotor function early in the acute phase of the injury which was resolved by the end of the acute phase. Locomotor performance in SCI mice was completely recovered at 4 wpi and in the chronic phase of the lesion. All mice were able to move freely without paralysis or major impairment in coordination and locomotor function when the affective-related behavioral tests were performed.

Two-way ANOVA analysis indicated significant effects of time (*p* < 0.01) and surgery (*p* < 0.01) factors and significant interaction for time × injury (*p* < 0.01). Post-hoc analysis showed that mild SCI induced a significant decrease in BMS scores until 3 wpi when compared to naïve and control mice (*p* < 0.05). Mice that underwent the sham procedure only showed a decrease in BMS score at 1 wpi when compared to the naïve group. After 3 wpi, no significant differences in BMS scores were found between the three experimental groups (*p* > 0.05).

Altogether, these findings indicate that mild SCI induced development of long-lasting thermal hyperalgesia (up to 12 wpi) and mechanical allodynia (up to 8 wpi) without motor impairments that could interfere with further behavioral assessment.

### 2.2. Transient Upregulation of Spinal Sensitization-Related Algogens in the Acute Phase of SCI

**Figure 2:**
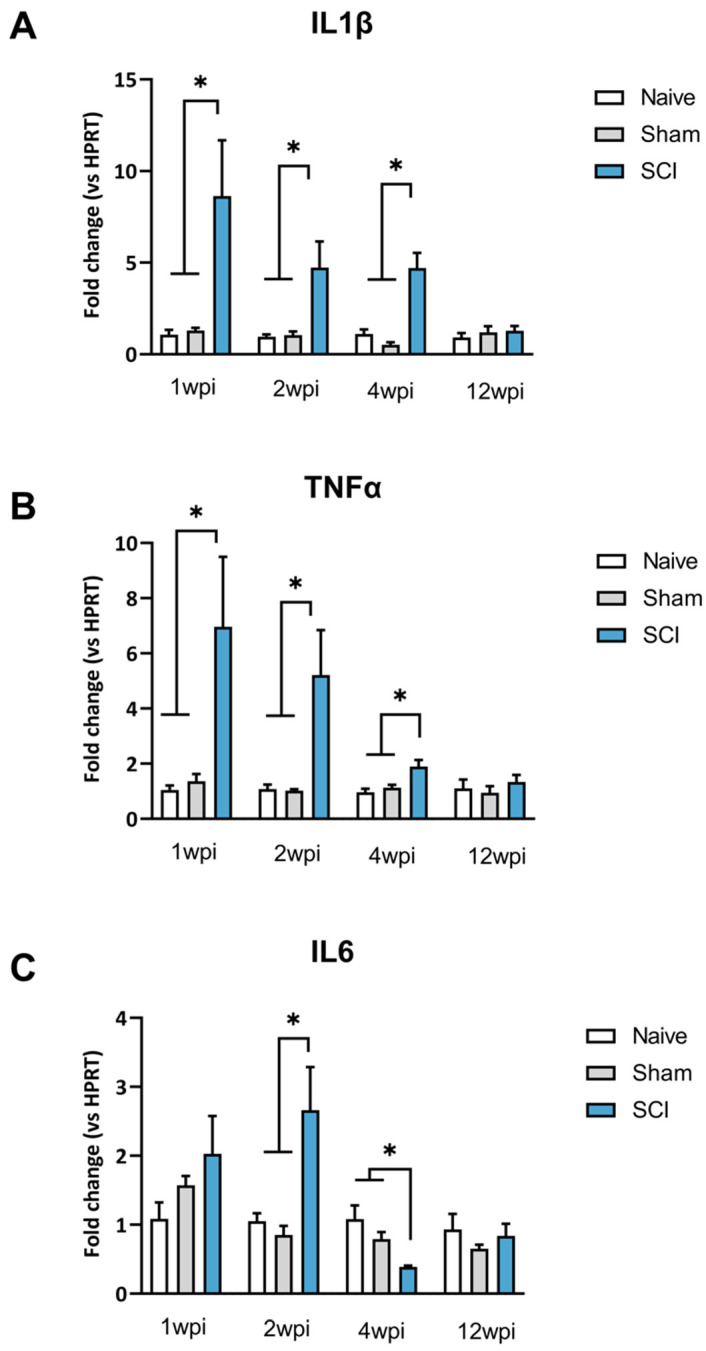
Spinal cytokine expression after mild spinal cord injury (SCI). IL1β (**A**), TNFα (**B**), IL6 (**C**) showed expression changes compared to naïve and sham mice during the acute phase of SCI (1,2,4 wpi) in T8–T9 segments of the spinal cord. No expression changes were detected during the chronic phase of the SCI (12 wpi). Results are the mean ± SEM. Experimental groups: Naive (n = 4–6), Sham (n = 5–6) and SCI (n = 5–6). * *p* < 0.05 (one-way ANOVA followed by Tukey’s post-hoc test). IL1β, Interleukin 1β; TNFα; Tumor necrosis factor-α; IL6, Interleukin-6; SCI, spinal cord injury; wpi, weeks post injury.

After mild SCI, mice showed a transient increased expression of spinal proinflammatory cytokines during the acute phase. In the chronic phase, the expression of proinflammatory cytokines was comparable to the expression levels of uninjured control mice (naïve and sham groups) (Figure 2).

One-way ANOVA analysis of IL1β expression revealed significant group differences at 1 wpi (*p* = 0.0257), 2 wpi (*p* = 0.0179) and 4 wpi (*p* = 0.0002). However, a lack of group differences was detected at 12 wpi *p* = 0.6463) (Figure 2A). Specifically, post-hoc analysis indicated that IL1β expression was significantly increased in comparison to both sham and naive groups up to 4 wpi (Figure 2B).

One-way ANOVA analysis of TNFα expression revealed group differences during the acute phase of SCI. Particularly, significant group differences were shown at both 1 wpi (*p* = 0.0217), 2 wpi (*p* = 0.0321) and 4 wpi (*p* = 0.0063). Post-hoc analysis revealed that TNFα was significantly overexpressed in the spinal cord of SCI mice when compared to control groups. In contrast, during the chronic phase of SCI at 12 wpi there was a lack of significant differences between groups (*p* = 0.6002) (Figure 2B).

Concerning IL-6, one-way ANOVA analysis revealed no group differences at 7 dpi (*p* = 0.2830) but significant differences at 2 wpi (*p* = 0.0243) and 4 wpi (*p* = 0.0041). Post-hoc analysis showed a significantly increased IL6 expression after 2 wpi and a significant decrease at 4 wpi compared to naïve and sham mice (Figure 2C). These results indicate significant fluctuations of IL-6 expression during the acute phase after SCI. Later, similarly to IL1β and TNFα, a lack of significance was detected at 90 dpi (Figure 2C).

### 2.3. Long-Lasting Disturbances on Hedonic and Social Behaviors Associated with Mild Spinal Cord Injury-Induced Neuropathic Pain

Several behavioral tests were used to investigate if the SCI-derived neuropathic pain could induce hedonic and social alterations at the acute phase (4 wpi) and at the chronic phase (12 wpi).

**Figure 3:**
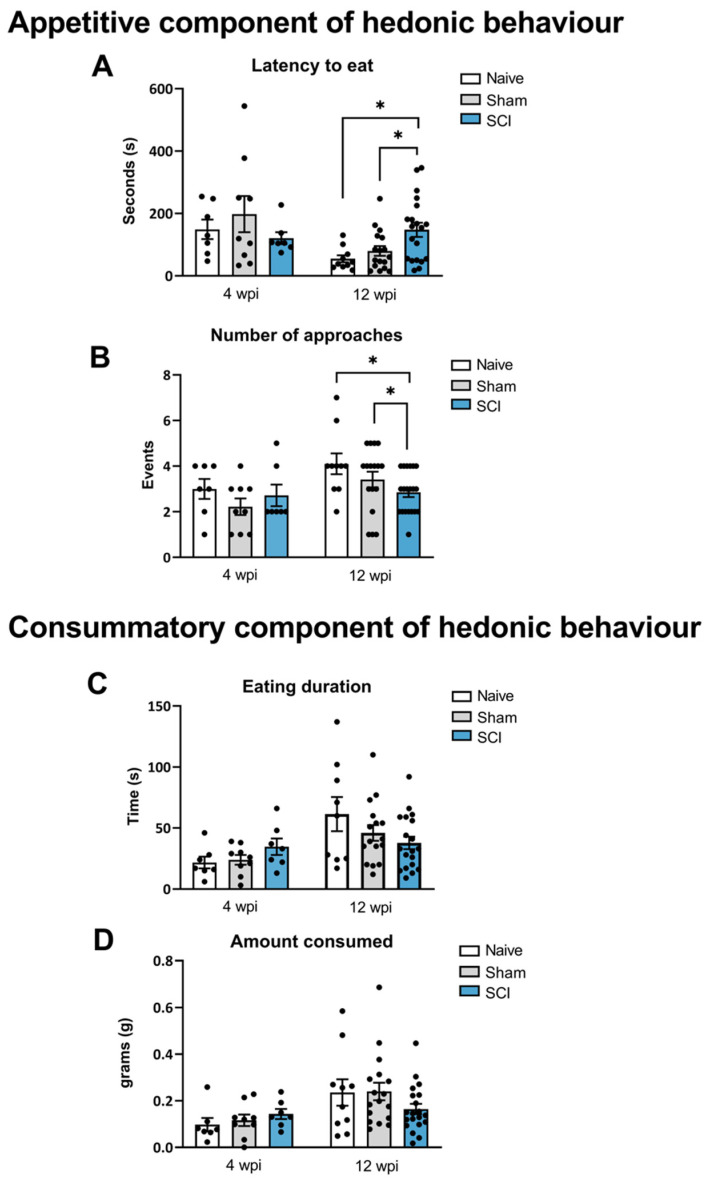
Reward-Seeking Behavior (RSB) test after mild spinal cord injury (SCI). Alterations in the hedonic state of the SCI-injured mice were detected at 12 wpi. Latency to eat (**A**) and approaches to eat (**B**) describe the appetitive component of the hedonic behavior whereas the eating duration (**C**) and the amount consumed (**D**) report the consummatory component of hedonic behavior in the RSB test. Results are the mean ± SEM.). * *p* < 0.05 (one-way ANOVA followed by Tukey’s post-hoc test). SCI, spinal cord injury; wpi, weeks post injury.

The Reward-Seeking Behavior (RSB) test is used to evaluate the appetitive and consummatory component of the hedonic state of the animals. During the acute phase (4 wpi), one-way ANOVA analysis revealed no significant group differences in any of the parameters studied: latency to eat reward (*p* = 0.4561), number of approaches to reward (*p* = 0.4131), total time eating (*p* = 0.2056) and g of consumption (*p* = 0.4814) (Figure 3A–D).

During the chronic phase (12 wpi), one-way ANOVA analysis revealed significant group differences in parameters such as latency to eat the reward (*p* = 0.0054) and number of approaches to the reward (*p* = 0.04), which are both descriptive of appetitive behavior. Specifically, post-hoc analysis indicated that mice with spinal cord lesions spent significantly more time until the first bite or licking of the chocolate piece when compared to naïve and sham groups (*p* < 0.05). These animals also showed a significant decrease in the number of approaches to the reward in comparison with control groups (*p* < 0.05) (Figure 3A,B). On the other hand, no effects on consummatory behavior were detected since no significant differences were found in eating duration at 4 wpi (*p* = 0.2056) or 12 wpi (*p* = 0.133) post-injury or in the amount consumed at 4 wpi (*p* = 0.4814) or 12 wpi (*p* = 0.2134) (Figure 3C,D).

These results may suggest that SCI-induced neuropathic pain induces suppression of the appetitive component of hedonic behavior at the chronic phase of SCI, evidenced by the decreased motivation to reach highly palatable food.

**Figure 4:**
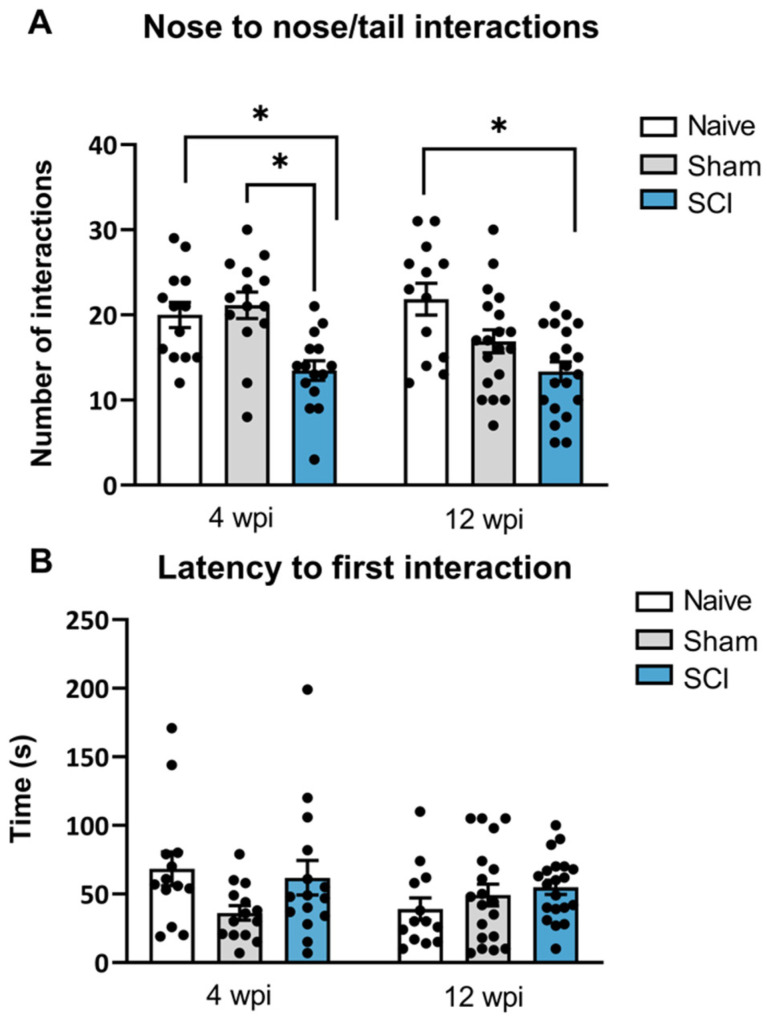
Social interaction test after mild spinal cord injury (SCI). Social behavior deficits were detected in SCI mice as they showed a decreased number of nose-to-nose/tail interactions (**A**) with a conspecific mouse both at 4 wpi and 12 wpi. No differences were obtained for the latency of first interaction with conspecific mice (**B**) Results are the mean ± SEM. * *p* < 0.05 (one-way ANOVA followed by Tukey’s post-hoc test). SCI, spinal cord injury; wpi, weeks post injury.

A social interaction test was used to measure the preference or motivation for initiating social interactions with a conspecific mouse. Social behavior deficits were observed both during the acute phase (4 wpi) and the chronic phase (12 wpi). Specifically, at 4 wpi, one-way ANOVA showed significant differences between groups in direct interactions (*p* = 0.0005). Post-hoc analysis indicated that SCI mice significantly decreased the amount of direct interactions nose-to-nose and nose-to-tail with the conspecific mouse, when compared to sham and naïve mice. Similarly, at 90 dpi, significant group differences were found in the direct interactions as shown by one-way ANOVA analysis (*p* = 0.0008) (Figure 4A). Particularly, post-hoc analysis showed that SCI-injured mice also showed a significantly decreased number of direct contacts with the conspecific when compared to the naïve group.

No differences between groups were found in the latency to first interaction at 8 wpi (*p* = 0.0955) or 12 wpi (*p* = 0.3244). Although the latency to first interaction did not reveal significant differences, direct contact interaction is a strong indication of social motivation or social affiliation. Therefore, these findings indicate that spinal cord-injured mice experiencing central neuropathic pain had a decreased social motivation to interact in the acute phase of the injury which persisted until the chronic phase of the injury.

### 2.4. No Anxiety-like Behavior Development Was Evidenced after Mild Spinal Cord Injury-Induced Neuropathic Pain

**Figure 5:**
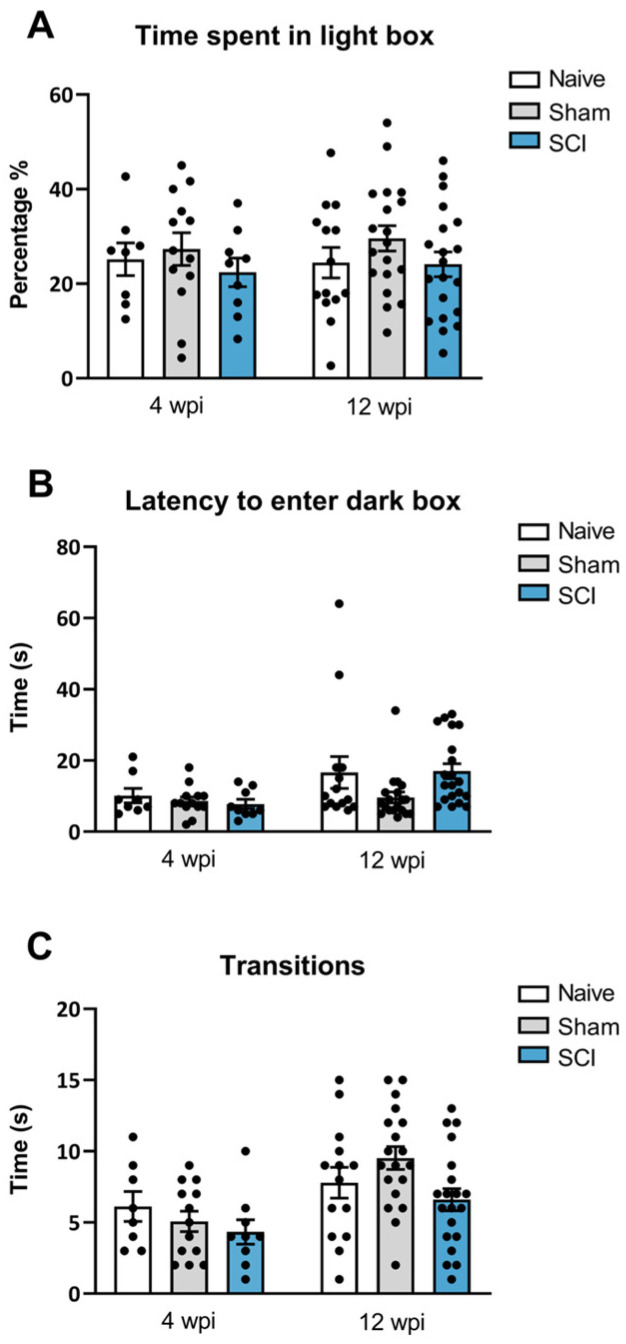
Dark and light box test after mild spinal cord injury (SCI). Time spent in the light box (**A**), the latency to enter the dark box (**B**) and the transitions (**C**) between boxes were evaluated. SCI mice did not show anxiety-like behavior either at 4 wpi or 12 wpi. The results are the mean ± SEM. SCI, spinal cord injury; wpi, weeks post injury.

In addition to the behavioral analysis explained above, anxiety-like behavior was also evaluated at both phases of the mild SCI. In contrast to social and motivational behaviors analysis, no anxiety-like behavior was found after mild SCI. In the dark/light box test performed at 8 wpi, ANOVA analysis revealed no significant group differences either for the percentage of time animals spent in the light compartment (*p* = 0.5861) or in the latency to go to the dark chamber (*p* = 0.569) (Figure 5B). Similarly, at 12 wpi, ANOVA analysis indicated no significant differences between groups either for the percentage of time animals spent in the light compartment chamber (*p* = 0.294) (Figure 5A) or in the latency to this dark zone (*p* = 0.085) (Figure 5B). Furthermore, no significant effects were detected on the number of transitions between dark and light compartments either at 28 (*p* = 0.3985) or 90 days post-injury (*p* = 0.0512) (Figure 5C), suggesting no locomotor deficit at any time point of evaluation. Overall, these findings indicate that mild spinal cord-injured mice did not develop anxiety-like behavior during the experimental period, either at the end of the acute injury phase or at the chronic phase.

### 2.5. Significant Upregulation of Central Sensitization-Related Algogens in Supraspinal Structures up to 12 Weeks Post-Injury

To explore whether the affective behavior alterations were co-occurring with expression of central sensitization algogens, several supraspinal structures were evaluated. IL1β, TNFα and IL6 were evaluated in the medial prefrontal cortex, nucleus acumens and amygdala, structures known to play pivotal roles in motivational, social or anxiety behavioral processes.

**Figure 6:**
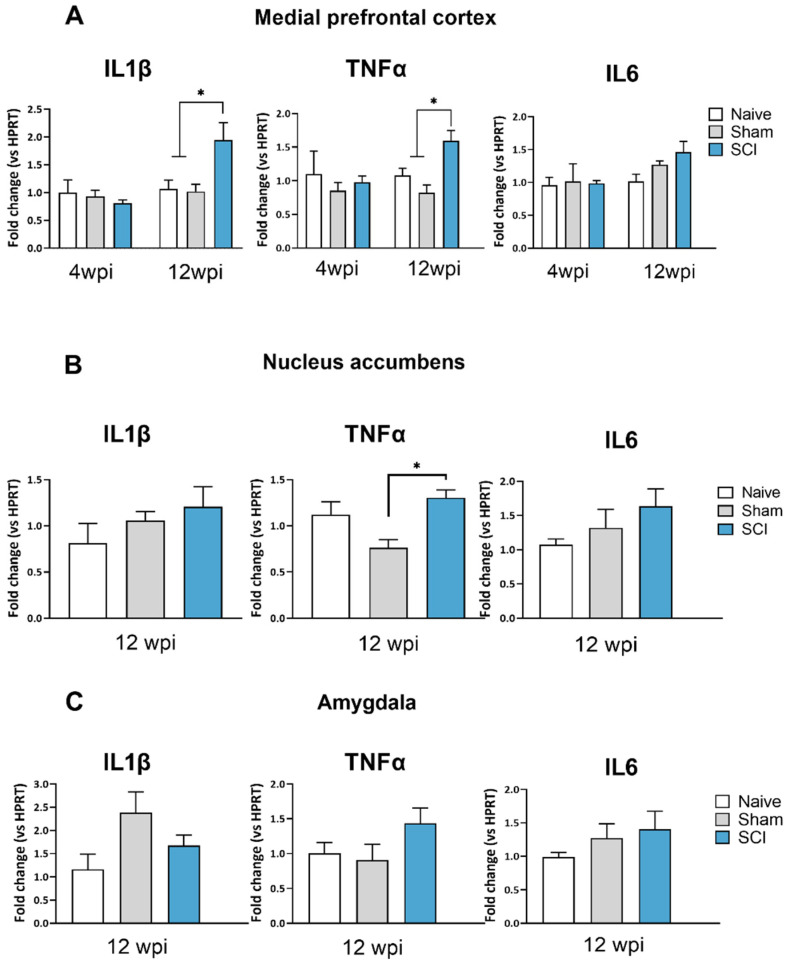
Supraspinal cytokines expression after mild spinal cord injury (SCI). SCI mice showed an increase of IL1β and TNFα in the medial prefrontal cortex (**A**) at 12 wpi, an increase in TNFα in nucleus accumbens (**B**) at 12 wpi and no cytokine expression changes in the amygdala (**C**). The results are the mean ± SEM. Experimental groups: Naive (n = 4–6), Sham (n = 5–6) and SCI (n = 5–6). * *p* < 0.05 (one-way ANOVA followed by Tukey’s post-hoc test). IL1β, Interleukin 1β; TNFα; Tumor necrosis factor-α; IL-6, Interleukin-6; SCI, spinal cord injury; wpi, weeks post injury.

In the mPFC, ANOVA analysis of IL1β and TNFα expression revealed significant group differences at 90 dpi (IL1β, *p* = 0.0002; TNFα, *p* = 0.032) but a lack of significance at 28 dpi (IL1β, *p* = 0.6463; TNFα, *p* = 0.66). Specifically, at the chronic phase, spinal cord-injured mice showed significant upregulation of both IL1β and TNFα expression when compared with sham or naïve animals (Figure 6A). For IL6, the ANOVA analysis indicated no differences between groups at 4 wpi (*p* = 0.9696), although a tendency towards statistical significance was observed at 90 dpi (*p* = 0.0714) (Figure 6A).

In the nucleus accumbens, one-way ANOVA analysis also revealed significant group differences of TNFα expression between groups (*p* = 0.0179) at 12 wpi. Post-hoc analysis showed that TNFα was overexpressed when compared to sham mice. In contrast, results indicated that neither IL1β (*p* = 0.3882) nor IL6 (*p* = 0.232) showed differences between groups at 8 wpi (Figure 6B).

Finally, in the amygdala, no significant group differences were observed in IL1β, TNFα and IL6 at 12 wpi (IL1β, *p* = 0.6463; TNFα, *p* = 0.0243; IL6 *p* = 0.032)

### 2.6. Significant Microgliosis, Astrogliosis and CX3CL1/CX3CR1 in ACC Compartments of mPFC at Chronic SCI Phase

To elucidate whether gliosis and CX3CL1/CX3CR1 expression in mPFC and its anterior cingulate cortex (ACC) compartments are involved in developing affective nonreflexive pain responses, a new set of SCI-chronic animals was prepared. After ensuring these new animals showed long-lasting reflexive and nonreflexive pain responses induced by SCI (see Supplementary Figures S1 and S2), Western blot and immunohistochemical analyses were performed.

**Figure 7:**
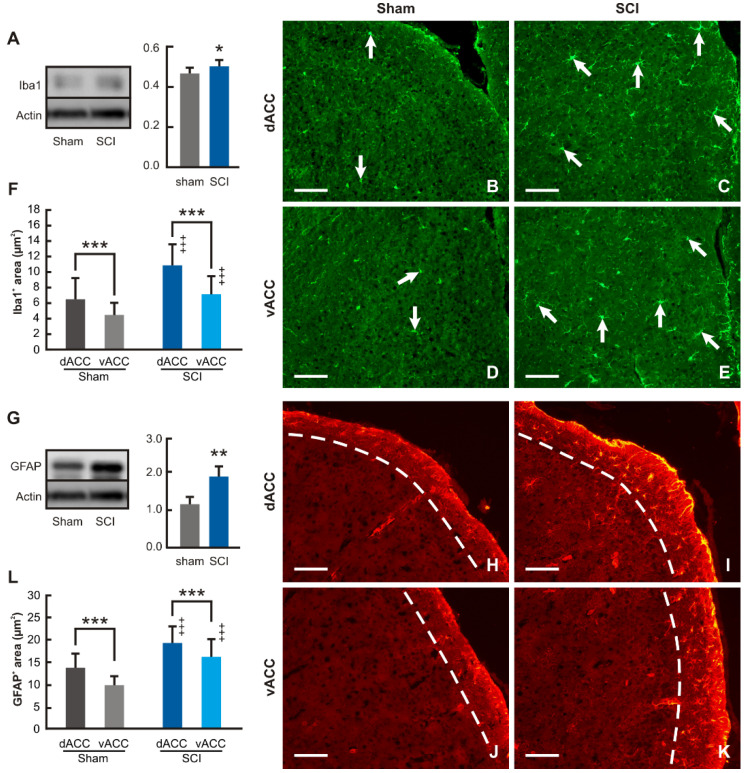
Western blot and immunohistochemical analyses of microgliosis and astrogliosis in ACC. (**A**) Results of Western blot analysis revealed significantly increased levels of Iba1 protein in the mPFC samples removed from SCI- than sham-operated mice (n = 6 for each group; * *p* < 0.05 vs. Sham). (**B**–**E**) Representative pictures illustrating Iba1 immunostaining for detection of activated microglial cells in the dorsal and ventral compartments of ACC (dACC and vACC, respectively) from sham- (Sham) and SCI-operated (SCI) mice. More activated microglial cells with typical morphology (arrows) were found throughout both dACC and vACC profiles in SCI- than in Sham-operated mice. Scale bars = 100 µm. (**F**) Image analysis detected significantly higher proportions of Iba1 immunostaining in dACC than vACC in both sham- and SCI-operated animals (n = 6 for each group; *** *p* < 0.001 vs. Sham). In addition, SCI induced the increase of Iba1 immunofluorescence proportion in both dorsal and ventral compartments of ACC compared with samples from sham-operated mice (^+++^ *p* < 0.001 vs. Sham). (**G**) Results of Western blot analysis revealed significantly increased level of GFAP protein in the ACC samples of SCI- than sham-operated mice (n = 6 for each group; ** *p* < 0.01 vs. Sham). (**H**–**K**) Representative pictures illustrate the increased proportion of GFAP immunostaining predominantly in lamina-I delineated by the dashed lines in both dACC and vACC compartments of SCI- compared to those from sham-operated mice, confirming the results of Western blot. Scale bars = 100 µm. (**L**) Significantly higher proportion of GFAP immunostaining in lamina-I of dACC than vACC compartments was found in both sham- and SCI-operated mice (n = 6 for each group; *** *p* < 0.001 vs. Sham). The results of image analysis also demonstrated significantly increased proportions of GFAP immunofluorescence in both dACC than vACC of SCI- than sham-operated mice (^+++^ *p* < 0.001 vs. Sham). Full-length blots are presented in Supplementary Figure S3.

Western blot analysis revealed significantly increased levels of Iba1 protein in the samples of ACC from SCI-operated compared with sham-operated mice for 12 wpi (Figure 7A). Iba1 immunostained microglial cells were found throughout the cortical profile of dACC and vACC compartments in mPFC of both sham- and SCI-operated mice. Moreover, many Iba1 immunopositive microglial cells in the ACC of SCI-operated mice had enlarged bodies and sent off distinct processes (Figure 7B–E). Based on very similar results of Iba1 quantification obtained in individual cortical laminae, we expressed the results of image analysis of Iba1 as the ratio of Iba1 in dACC and vACC associated with activation of microglial cells. In line with the results of the Western blot, the proportion of Iba1 immunostaining increased substantially in both dACC and vACC of SCI-operated animals surviving for 12 wpi when compared with those from sham-operated animals, reflecting an increase in the activation of microglial cells. In addition, the level of microglial cell activation was always higher in dACC than vACC in both sham- and SCI-operated mice (Figure 7F).

Western blot analysis and immunostaining of GFAP was used for detection of reactive astrocytes in dACC and vACC compartments. Western blot showed significantly increased levels of GFAP protein in the ACC samples of SCI-operated mice for 12 wpi when compared with the sham-operated control group of animals (Figure 7G). GFAP immunostaining used for the detection of reactive astrocytes was observed predominantly only in lamina-I of the mouse dACC and vACC compartments (Figure 7H–K). Therefore, image analysis of GFAP immunostaining to estimate reactive astrocytes was limited to lamina-I of dACC and vACC. The results of the measurements revealed a significantly increased proportion of GFAP immunostaining in the lamina-I of dACC and vACC from SCI- in comparison to sham-operated mice. Moreover, similar to microglia activation, astrocyte reactivity was always higher in dACC than vACC in both sham- and SCI-operated mice (Figure 7L). In summary, image analysis of GFAP immunostaining thus showed increased astrocyte reactivation in the lamina-I of both dACC and vACC after chronic SCI.

**Figure 8:**
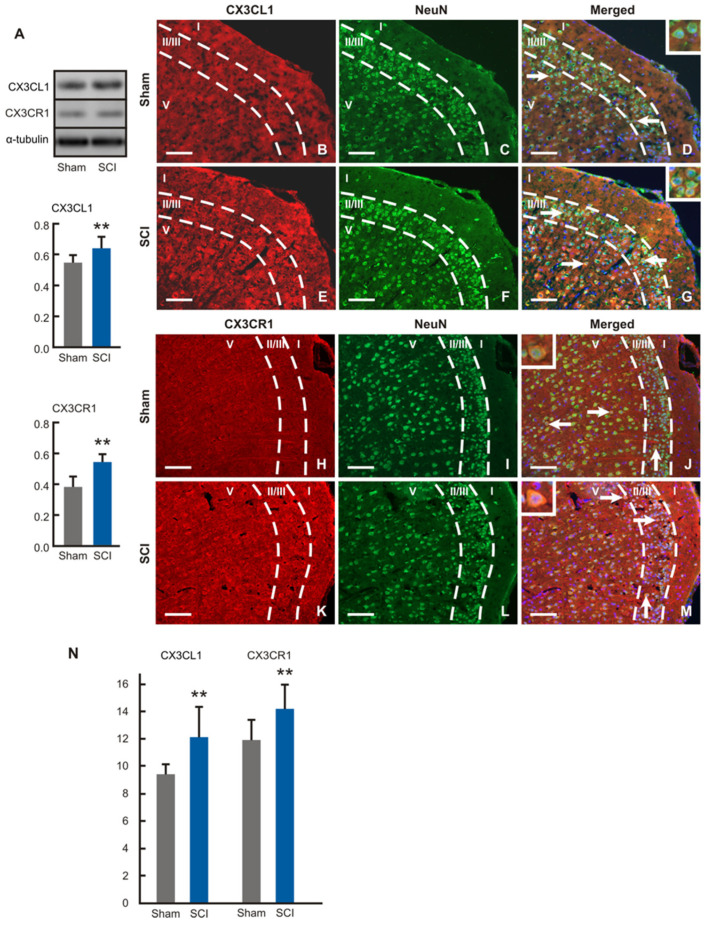
Western blot and immunohistochemical analyses of CX3CL1 and CX3CR1 in ACC. (**A**) Western blot analysis demonstrated significantly increased levels of both CX3CL1 and CX3CR1 proteins in the ACC samples removed from SCI- than sham-operated mice (n = 6 for each group; ** *p* < 0.01 vs. Sham). (**B**–**M**) Representative pictures illustrating double immunostaining for detection of CX3CL1 (**B**–**G**) or CX3CR1 (**H**–**M**) and NeuN showing neuronal localization of this chemokine and its receptor (arrows and insets) in laminae-II/III and lamina-V of ACC. CX3CL1 or CX3CR1 antibody binding was visualized by TRITC-conjugated and binding of NeuN antibody by FITC-conjugated secondary antibody. In addition, merged pictures contain blue decorated nuclei stained by Hoechst 33,342. Scale bars = 100 µm. (**N**) Image analyses of immunofluorescence intensities confirmed increased levels of CX3CL1 and CX3CR1 proteins in the ACC neurons of SCI- compared with sham-operated mice (n = 6 for each group; ** *p* < 0.01 vs. Sham). Full-length blots are presented in Supplementary Figure S4.

Protein levels of CX3CL1 and CX3CR1 were significantly higher in the ACC samples removed from SCI- than sham-operated mice (Figure 8A). Surprisingly, immunostaining of CX3CL1 and CX3CR1 was detected only in the neurons throughout II/III and V laminae in dACC and vACC of both sham- and SCI-operated mice. The CX3CL1 and CX3CR1 immunopositivity was localized in the pyramidal and nonpyramidal neurons dispersed in the cortical laminae (Figure 8B–M). Measurement of immunofluorescence intensities showed significantly increased expression of both CX3CL1 and CX3CR1 proteins in the cortical neurons of SCI-operated compared with sham control animals (Figure 8N).

## 3. Discussion

The findings obtained in this study indicated that after mild SCI, female CD1 Swiss mice developed chronic CNP up to 12 weeks post-injury. Specifically, SCI animals showed thermal hyperalgesia up to 12 weeks after injury and mechanical allodynia up to 8 weeks. While it is true that female mice developed chronic reflexive pain responses, it is also true that animals showed increased paw withdrawal thresholds during the chronic SCI phase, when compared with acute phase. These findings suggest that the animals showed a progressive decrease of reflexive pain responses along the experimental period up to late-chronic phase. However, it is difficult to elucidate whether these increased withdrawal thresholds would be associated with lower hypersensitivity or, in contrast, may be attributable to other reasons such as habituation to test or other behavioral disturbances that may mask pain responses. Considering that evoked pain response evaluation may be affected by some of these limitations [35,36], during the last decade it has been also proposed to evaluate the nonreflexive pain responses, which involve supraspinal structures associated with pain pathways. That is, pain is not just a reflex, but rather a perceptual experience with powerful emotional and motivational components [35], and these nonreflexive or operant responses could even provide more clinical concordant results in humans [35]. In this regard, our results showed that although reflexive pain responses decreased over time in the SCI animals, the affective/motivational nonreflexive pain responses were significant mainly in the chronic phase. Indeed, it is widely known that SCI patients usually develop long-term emotional disorders [37,38,39]. Although these behavioral disturbances have been described in SCI animal models [21,22,40,41], most of them have been evaluated by classical behavioral testing after moderate or severe spinal cord contusion, and the interpretation of behavioral tests could be misled by motor deficits. In the present study, nonreflexive pain responses were evaluated by means of suitable tests that evaluated non-operant motivational tasks such as social interaction [42] and reward-seeking behavior tests [43]. Social interaction tests were used to evaluate social responses that mice exhibit when exposed to a conspecific. The preference for initiating social interactions was evaluated as a measure of sociability. The Reward-Seeking Behavior (RSB) test was used to first evaluate the motivation to pursue and approach a rewarding stimulus, in this case a white chocolate pellet (appetitive component), and then the hedonic response to it (consummatory component).

Mice subjected to SCI showed decreased preference to socially interact with a conspecific mouse at both acute and chronic phases of SCI as shown by the decrease in direct contacts. Moreover, an alteration in the appetitive component of hedonic behavior was detected only at the chronic phase of SCI. Particularly, mice that underwent SCI showed a decreased motivation to approach highly palatable food, but the consummatory behavior that follows was unaltered, evidencing a clear anhedonic state. These results are very interesting because while severe SCI has been associated with some emotional and cognitive alterations, mild SCI can result in no major affective disturbances [24]. So, the present work provides for the first time behavioral approaches that are more sensitive to detecting affective disturbances in a SCI mice model that were undetected in other studies. For the anxiety-like behavior, the results are consistent with previous studies that stated no anxiety disturbances after mild SCI [24]. Specifically, the D/L box test indicated that mild spinal cord injured mice did not develop anxiety-like behavior during the experimental period, either at the end of the acute injury phase or in the chronic phase. These results may not be attributable to locomotor disturbances since no differences on the number of transitions were observed during the dark/light box test at any time point of evaluation. Moreover, our findings indicated that mild spinal cord-injured mice showed no major locomotor disturbances, since the BMS rating scores were higher than 6 at 7 days after injury and reached about 9 before the chronic phase started. These results indicate that SCI female mice were able to freely move without paralysis without hampering the behavioral evaluation tests. Together with the findings explained above, a suitable model to study central chronic neuropathic pain-like behaviors without major locomotor disturbances was obtained.

Remarkably, these reflexive and nonreflexive pain responses associated with neuroinflammation-related molecular expression resulted from the spinal and supraspinal structures analyses. The reflexive pain responses observed during the SCI acute phase were consistent with the upregulation of IL1β, TNFα and IL6 in the spinal cord, which are known to be algogenic biomarkers that have been reported to be involved in central neuropathic pain development [44,45]. However, these upregulations were transient and no changes in their expression were detected during the chronic SCI phase. The transient proinflammatory cytokine upregulation during the acute SCI phase has been already described and is related to the secondary injury process in SCI pathophysiology [46,47]. It is known that this transient nervous system inflammatory response also targets downstream pathways which can disturb neural function such as the excitation of the N-methyl-D-aspartate (NMDA) receptor triggering neuronal hyperexcitability and central sensitization. Indeed, chronic inflammation can lead to a permanent restructuring of central neurotransmitter pathways and to the transition from acute to chronic pain, even after the acute inflammatory response has dissipated [48]. The latter may thus explain the lack of upregulation of proinflammatory algogens detection during the SCI chronic phase.

On the other hand, while no upregulation of proinflammatory cytokines was detected in the spinal cord during the chronic phase, results revealed neuroinflammatory patterns in supraspinal structures that play pivotal roles in affective behaviors (or nonreflexive pain responses). The results showed a TNFα upregulation in both mPFC and NAcc and IL1β upregulation in mPFC. In contrast, no neuroinflammatory response was detected in the amygdala. Previous studies in rodents have linked supraspinal neuroinflammatory processes with behavioral disturbances after SCI [22,41,48], specifically focusing on the hippocampus, amygdala and cerebral cortex. The present study was focused on mPFC and NAcc, interconnected limbic system structures associated with affective functions specifically involved in social and motivational behaviors which are lesser studied in animal models of SCI. mPFC plays a crucial role in mouse social behavior and disturbed excitatory and inhibitory balance in this supraspinal structure induced social behavior dysfunction [49]. As for NAcc, it is known that it plays a key role in brain reward processes, including reward-seeking behaviors [34]. Indeed, several pieces of evidence suggest that pain, in particular long-term pain, impairs several aspects of reward and affective processing. Interestingly, evidence is found in the literature describing the affective component of pain. Anhedonia was associated with chronic pain [5,50,51]. Decreased reward sensitivity and/or decreased motivation was observed in rats with neuropathic pain [52]. Impaired decision-making based on reward and punishment was reported in patients with chronic back pain and complex regional pain syndrome (CRPS) [53] as well as in an animal model of arthritic pain [54]. Finally, impaired operant learning of pain sensitization and habituation was found in fibromyalgia patients [55]. Altogether, these findings suggest that both mPFC and NAcc alterations may be associated with chronic pain, triggering motivational and social deficit behaviors, as seen in the spinal cord-injured animals of the present study.

Although the association between pain and supraspinal neuroinflammatory processes have been described so far, the mechanisms underlying SCI-mediated effects in the brain remain speculative. To gain mechanistic insights, considering that mPFC is involved in the development of affective behavioral changes during chronic neuropathic pain [56], the present work was complemented by a further detailed analysis of glial cell activation and regulation of chemokine CX3CL1 and its receptor CX3CR1. The rodent mPFC is subdivided into four main divisions, the medial agranular, anterior cingulate, prelimbic, and infralimbic cortex [57,58]. Moreover, the ACC of rodents can be further subdivided into dorsal (dACC) and ventral (vACC) compartments described also as cortical area 24b and 24a, respectively [58], with dACC involved in behavioral actions leading to rewards [59] and vACC in both autonomic visceromotor and cognitive functions [60].

Increased activation of microglial cells and reactivity of astrocytes in the samples of mPFC removed from SCI- and sham-operated mice are in line with previously published results obtained in other experimental models of neuropathic pain [61,62]. In addition, image analysis revealed a higher level of microglia activation and astroglial reactivity in dACC than vACC, and their increase after SCI compared with the sham operation. Activated microglial cells and reactive astrocytes regulate neuronal homeostatic plasticity and have a crucial role in the neuronal excitation, reorganization of neuronal circuits and synaptic remodeling [63,64]. This, in the case of greater activation of glial cells in dACC, may be related to the observed changes in reward-seeking behavior after SCI.

There is a growing body of evidence that chemokines and their receptors are upregulated in the brain regions critical for the affective state of neuropathic pain [65,66]. Chemokine CX3CL1 and its receptor CX3CR1 have been frequently studied in the spinal cord of various neuropathic pain models. Whereas CX3CL1 was found in the neurons [67], CX3CR1 was mainly detected in microglial cells [68,69]. These results suggested that this chemokine axis is used by spinal cord neurons to communicate with microglial cells and modulate their activation [70,71,72,73]. In this study, we found increased expression of both CX3CL1 and CX3CR1 in ACC neurons of spinal cord injured animals. Chemokines/chemokine receptors signaling axis might have a neuromodulatory or neuroprotective role in the brain structures after various types of pathological conditions [74]. Specifically, several experimental proofs have been made for chemokine CX3CL1 and its receptor CX3CR1 [75,76,77]. Based on our results of neuronal detection and its increase after SCI, we can speculate that the CX3CL1/CX3CR1 signaling axis could also be involved in neuromodulation reactions in ACC associated with affective nonreflexive pain responses during the SCI chronic phase. However, further experiments are needed to prove the neuromodulatory or neuroprotective effect of increased neuronal levels of CX3CL1/CX3CR1 in ACC induced by chronic SCI.

## 4. Materials and Methods

### 4.1. Animals

Five-week-old female ICR CD1 mice (19–26 g) were purchased from Charles River Laboratories (Saint-Germain-Nuelles, France) and Janvier Laboratories (Le-Genest-Saint-Isle, France, France). Mice were housed in groups of five in temperature- and humidity-controlled laboratory conditions (21 ± 1 °C, 55 ± 10%) maintained with food and water ad libitum. All mice were allowed to acclimatize to the facility rooms prior to initiating any behavioral or surgical procedures, which were performed during the light cycle. All experimental procedures and animal husbandry were conducted following the ARRIVE guidelines and according to the ethical principles of the I.A.S.P. for the evaluation of pain in conscious animals and the European Parliament and the Council Directive of 22 September 2010 (2010/63/EU) and were approved by the Animal Ethics Committee of the Parc Científic of Barcelona. In agreement, maximal efforts were made to reduce the suffering and the number of mice used.

### 4.2. Experimental Design

This study was aimed at evaluating the nociceptive, affective, and biochemical effects of a mild SCI, in both acute and chronic phases of the lesion.

To this end, CD-1 WT female mice were subjected to a mild SCI and nociceptive behavior (thermal hyperalgesia and mechanical allodynia) was measured with von Frey test and plantar test, respectively, throughout the acute phase (1, 2, 3, 4 weeks post injury; wpi) and during the chronic phase (60 and 90 wpi). The affective state of the animals was evaluated both in the acute (4 dpi) and chronic phase (8 dpi) of the spinal cord injury. The reward seeking behavior (RSB) test was used to evaluate the hedonic behavior towards a rewarding stimulus. The social interaction test was used to evaluate social preference or motivation towards a conspecific. Finally, the light and dark box tests were performed to investigate anxiety-like behavior. Independent experimental groups were used to avoid stress-related behavior and habituation to the test.

To study the neuroinflammatory processes occurring centrally, the spinal cord at the site of the injury (T8–T9 level) was dissected at 1, 2, 3, 4, 8, 12 wpi. To evaluate the neuroinflammatory consequences after a mild SCI in supraspinal structures, several brain regions were dissected at 4 wpi and 12 wpi. Following the Paxinos atlas, medial prefrontal cortex, nucleus accumbens and amygdala were carefully dissected. mRNA expression changes of several proinflammatory cytokines in the before mentioned structures were measured with quantitative real time PCR.

Lastly, microglial and astroglial changes and fractalkine expression and its receptor were evaluated in the anterior cingulate cortex (ACC), a specific area of the mPFC with Western blot and immunohistochemical analyses at 12 wpi in an independent batch of animals.

### 4.3. Surgical Procedure

In order to obtain a central neuropathic pain mouse model without locomotor paralysis, mild spinal cord contusion was performed following procedures explained elsewhere [78,79,80]. Mice were anesthetized with sodium pentobarbital (50 mg/kg, i.p.), T8–T9 thoracic spinal cord segments were exposed via laminectomy and a contusion was induced by dropping 2 g of weight from 25 mm over to the exposed spinal cord. Then, the wound was closed and disinfected with povidone. To restore blood volume deficit after surgery, animals were given 0.5 mL of saline solution and kept in warmed cages with accessible food and water. As controls, sham and naïve mice were used. Sham surgery was performed by exposing the spinal cord at T8–9 levels via dorsal laminectomy but not contusioning.

Naïve mice did not receive any surgical manipulation. Prior to surgery, mice were randomly assigned to the three experimental groups (SCI injured, sham and naïve).

### 4.4. Locomotor Activity

The Basso Mouse Scale (BMS) test [81] was used to evaluate locomotor activity and performed as described elsewhere [78,79,80]. Concisely, animals were placed separately into a circular plastic open field (70 cm diameter × 24 cm wall height) and were allowed to move freely for 5 min. Meanwhile, the hindlimb movements were scored, considering their stepping, paw position, coordination, and trunk stability. The BMS score ranges from 0 (no hindlimb movement) to 9 (normal movement-coordinated gait).

### 4.5. Mechanical Allodynia and Thermal Hyperalgesia Assessments

Mechanical allodynia was quantified by assessing 50% withdrawal thresholds using a set of von Frey monofilaments (bending force range 0.04–2 g) following the up-down paradigm, as previously described [78,79,80,82]. Each filament was applied to the plantar surface of the mice for 2 s, then a thicker filament with an increased bending force range was successively applied until a nociceptive response was observed. Then, a thinner filament was applied. A total of 4 measures using this up and down procedures were scored. Nociceptive-like response was considered to be clear paw withdrawal, shaking or licking. Both hind paws were tested and averaged.

The mechanical threshold that produced 50% of responses was calculated using the Dixon formula: 50% paw withdrawal threshold (g) = [(10 (Xf + κδ)/10,000)], where Xf is the value (in logarithmic units) of the final von Frey filament used, k is a fixed tabular value for the pattern of positive/negative responses and d is the mean difference (in log units) between stimuli.

Thermal hyperalgesia was assessed by recording the hind paw withdrawal latency in response to radiant heat applied with the plantar test apparatus (IITC Life Science Inc., Los Angeles, CA, USA) as previously reported [83] and performed as described elsewhere [78,79,80]. Mice were allowed to acclimate on the tempered (29 °C) glass surface of the plantar cage exploration and major grooming activities ceased. Radiant heat source was positioned under the plantar surface of the hind paw with a time limit of 30 s to avoid skin damage. The withdrawal latencies for both hind paws were determined from the average of three separate trials, conducted at 5 min intervals. Both paws were evaluated since the SCI model results in a bilateral injury. Clear paw withdrawal, shaking or licking after heat stimulation were considered as nociceptive-like responses.

### 4.6. Light and Dark Box Test

The light and dark box (Panlab, Harvard apparatus, Cornellà de Llobregat, Spain) [84] consisted of a plexiglass box distributed into two compartments. The dimensions of the compartments were one third for the dark compartment (16 × 25 × 24 cm) and two thirds for the light compartment (25 × 25 × 24 cm). Mice were allowed to move from one compartment to the other through an opening (7 × 7 cm). No extra light source was used for the illumination, only the general room lighting (850 lx). Mice were placed individually in the light box facing the opening backwards. Transitions between the light and the dark box, time spent in each compartment and the latency time for the first passage from the light compartment to the dark one were recorded for 5 min with PPCWIN (Panlab S.L) software.

### 4.7. Reward-Seeking Behavior (RSB)

The basic test procedure was adapted from Merali [85] and was conducted as explained elsewhere [43]. Briefly, we analyzed the influence of pain on the hedonic behavior on a total of four measures: the latency and number of approaches to eat (“appetitive component”) and the amount and duration of the consumption (“consummatory component”). Latency to eat was defined as the time between the placement of the animal in the home cage and the first licking or biting of the chocolate piece. Amount of time was defined as the time spent by the mouse licking or biting the chocolate piece. The number of approaches refer to the approaches to the chocolate and further eating. Chocolate consumption was defined as the amount of chocolate consumed in grams and it was assessed by weighing the piece of chocolate before and after testing. Mice were habituated to palatable food 3 days before starting the experimental test to reduce food neophobia responses. Animals were given (~20 g) white chocolate fragments (Milkybar^®^, Nestle, S.A., Vevey, Switzerland) from a glass dish (4 cm diameter and 1 cm deep) placed in one corner of the home cage. On the test day, each animal had its own piece of chocolate (~2 g) that was placed in the glass prior starting the test. The behavioral test started as soon as each animal was placed opposite to where the chocolate is located. Each animal was recorded for 10 min. Furthermore, it is worth noting that to avoid neophobic behaviors against white chocolate all animals were habituated to the reward before testing.

### 4.8. Social Interaction Assessment

Social behavior tests were carried out in cages containing two grid enclosures (UGO basile), one empty and another with a stranger conspecific mouse. Based on Crawley’s social interaction test [86], two grid enclosures were placed at the opposite side of the arena. The explorer mouse was placed inside of the grid enclosures and the other was left empty. To avoid side preference bias, novel mice were placed to the right or left enclosure alternatively on consecutive assays. Two outcomes were scored for 10 min (direct interaction and latency to first direct interaction). Direct interaction is considered the most important and reliable parameter to assess social interaction and is the result of the total number of direct contacts (nose-to-nose and nose-to-tail) between the tested mouse and the enclosed novel mouse, subtracting the unspecific contacts (nose placement inside the grid in the empty enclosure). Latency to first direct interaction refers to the time until the tested mouse had their first direct interaction with the novel mouse.

### 4.9. RNA Extraction and Real Time Quantitative PCR

Mice were sacrificed by a lethal injection of pentobarbital (100 mg/kg). The spinal cord at the thoracic level (T8–T9), the mPFC (AP + 1.7, ML +/− 0.4, DV +1.8− > +3.0), NAcc (AP + 1.7, ML +/− 0.9, DV + 4.9) and amygdala (AP − 2.54, ML +/− 3, DV + 5) were dissected from the brains of females according to Franklin and Paxinos brain atlas. Samples (n = 3–6 per group) were fast frozen and then homogenated with TRI reagent with Ribopure Kit (Ambion/Applied biosystems, Foster City, CA, USA). RNA concentration was assessed with Nanodrop (Nanodrop Technologies, Rockland, DE, USA). Only RNA samples with 260/280 ratios between 1.8 to 2 were included.

Total RNA (1 ug) from each sample was reverse transcribed using the high-capacity cDNA reverse transcription kit (Applied Biosystems, Foster City, CA, USA).

For quantitative polymerase chain reaction (qPCR) with TaqMan^®^ gene expression assays, the following assays were used: Interleukin 1β (IL1β) (Mm00434228_m1), tumor necrosis factor α (TNFα) (m00443258_m1) and interleukin 6 (IL-6) (Applied Biosystems™). As endogenous control, Hypoxanthine-guanine phosphoribosyltransferase (HPRT) (Mm00446968_m1) (Applied Biosystems™) was used.

PCR reactions were run in triplicate in 384-well plates using a 7900HT Fast Real-Time PCR System (Applied Biosystems™). The PCR cycle conditions were 10 min at 95 °C, followed by 40 cycles of 15 s at 95 °C, and 60 s at 60 °C. All samples were run for each reference gene in the same plate to avoid between-run variations. Relative amount of target gene transcripts was calculated using the 2−ΔΔCt method as described previously [87].

### 4.10. Western Blot and Immunohistochemical Analyses

For Western blot analysis, mice were sacrificed with sodium pentobarbital (100 mg/kg, i.p.) and brains were removed and fast-frozen in liquid nitrogen, then stored at −65 °C until the time of Western blot analysis. A thick coronal section with near-perpendicular orientation to the cortical surface was carefully cut from each brain at a position approximately between 1.8 mm and 0.8 mm from the Bregma [88] and ACC was excised using manual microdissection under a stereomicroscope. Samples were homogenized in PBS containing 0.05% Tween 20 (PBS-TW20, pH 7.4) and protease inhibitors (LaRoche, Basel, Switzerland), and centrifuged at 15,000× *g* for 20 min at 4 °C. Protein concentration was measured in the tissue supernatant by Nanodrop ND-1000 (Thermo Scientific, Waltham, MA, USA) and normalized to the same levels. Each sample, containing 50 μg of protein, was separated by SDS-polyacrylamide gel electrophoresis and transferred to nitrocellulose membranes by electroblotting (BioRad, Hercules, CA, USA). The membranes were blocked with 1% BSA in PBS-TW20 for 1 h and incubated with rabbit polyclonal anti-Iba1 (1:1000; Wako, Fujifilm Wako, Neuss, Germany), anti-GFAP (1:250, Agilent DAKO, Santa Clara, CA, USA), anti-CX3CL1 (1:200; Abcam, Cambridge, UK) or anti-CX3CR1 (1:200; Abcam) overnight. Blots were washed in PBS-TW20 and incubated with peroxidase-conjugated anti-rabbit secondary antibodies (Sigma, St. Louis, MO, USA, 1:1000) at room temperature for 1 h. Equal loading of proteins was confirmed by β-actin (Iba1, GFAP) or α-tubulin (CX3CL1, CX3CR1) staining. Protein bands were visualized using the ECL detection kit (Merck KGaA, Darmstadt, Germany) on a LAS-3000 chemiluminometer reader (FujiFilm, Düsseldorf, Germany) and analyzed using densitometry image software.

For immunostaining, mice were perfused intraventricularly with 4% paraformaldehyde solution in phosphate-buffered saline (PBS, 10 mM sodium phosphate buffer, pH 7.4), and the brains were removed and post-fixed in 4% paraformaldehyde at 4 °C until analysis. The brains were washed in 20% phosphate-buffered sucrose (pH 7.4) for 48 h, and serial coronal sections (12 µm) through the prefrontal cortex area between 1.8 mm and 0.8 mm from the Bregma [88] were cut (Leica 1800 cryostat; Leica Microsystems, Wetzlar, Germany). The cryostat sections were collected on gelatin-coated microscopic slides, air-dried and processed for immunohistochemical staining. First, the sections were washed with PBS containing 0.05% Tween 20 (PBS-TW20) and 1% bovine serum albumin for 10 min, and then treated with 3% normal donkey serum in PBS-TW20 for 30 min. Then, the sections were incubated with rabbit anti-GFAP (1:250; Agilent DAKO, Santa Clara, CA, USA) or rabbit anti-Iba1 (1:100; Fujifilm Wako, Neuss, Germany) antibodies in a humid chamber at room temperature for 12 h. The binding of primary antibodies was visualized by secondary antibodies (FITC- or TRITC-conjugated, affinity purified goat anti-rabbit; 1:100; Jackson IR, Ely, UK) for 90 min at room temperature. To detect neuronal localization of CX3CL1 or CX3CR1, the sections were double immunostained at first by incubation with rabbit anti-CX3CL1 (1:200; Abcam, Cambridge, UK) or rabbit anti-CX3CR1 (1:200; Abcam, Cambridge, UK) at room temperature for 12 h, and with TRITC-conjugated and affinity purified goat anti-rabbit secondary antibody (1:100; Jackson IR, Ely, UK) for 90 min at room temperature. Then, the sections were immunostained with mouse monoclonal anti-NeuN antibody (1:500; Abcam, Cambridge, UK) for 240 min, and FITC-conjugated goat anti-mouse secondary antibody (1:100; Jackson IR, Ely, UK) for 90 min at room temperature. All sections of sham- and SCI-operated mice were immunostained simultaneously under the same conditions. Immunostained sections were rinsed, stained with Hoechst 33,342 to detect positions of the cell nuclei, and mounted in a Vectashield aqueous mounting medium (Vector Laboratories Inc., Burlingame, CA, USA). The control sections were incubated either without the primary antibodies or by substituting the primary antibodies with the IgG isotype. The control sections displayed no immunostaining. The sections were analyzed using a Nikon Eclipse NI-E epifluorescence microscope equipped with a Nikon DS-Ri1 camera and NIS-elements software (Nikon, Prague, Czech Republic).

### 4.11. Image Analysis

Image acquisition and image analyses were performed by a person blinded to experimental group.

At least 6 sections, separated from one another by an interval of about 60 μm of ACC, for each animal were selected for image analysis using the NIS-elements image analysis system (Laboratory Imaging Ltd., Prague, Czech Republic). The areas of interest were delimited in lamina-I (GFAP) and all laminae (Iba1) of the dorsal and ventral compartments of anterior cingulate cortex (dACC and vACC, respectively) that were defined according to Vogt and Paxinos [58].

The immunopositive areas for GFAP or Iba1 were detected by a thresholding technique after subtraction of background. The areas of immunostaining for GFAP or Iba1 were related to the areas of interest and expressed as the proportion of relative area (%) ± SEM.

Neuronal localization of CX3CL1 or CX3CR1 immunostainings was detected, and intensities of immunofluorescence were measured in red canal after subtraction of background according to our published protocol [89]. Intensities of CX3CL1 or CX3CR1 immunofluorescence were expressed as means ± SEM.

### 4.12. Statistical Analysis

Functional and molecular measurements were performed in a blinded manner using a code for both mice and samples. All data are expressed as the as mean ± SEM. The nociceptive and locomotor behavior (thermal hyperalgesia, mechanical allodynia and locomotor activity) were analyzed using two-way ANOVA (mixed effect model-REML) followed by Tukey’s post hoc when applicable. The affective evaluations performed with RSB, social interaction and light/dark box tests were analyzed using a one-way ANOVA followed by Tukey’s post hoc when applicable in each time point. Molecular results obtained by qPCR are expressed as fold change when compared to the control group (naïve mice) and analyzed using a one-way ANOVA followed by Tukey’s post hoc when applicable in each time point. The statistical analysis software used was GraphPad prism 9.0.

Molecular results obtained by Western blotting are expressed as the percentage of change when compared to the control group (Sham animals). Data of image analyses of GFAP or IBA1 immunostaining proportions as well as CX3CL1/CX3CR1 immunostaining intensities were compared between sham- and SCI-operated animals using a Mann–Whitney U-test (* *p* < 0.05, ** *p* < 0.01, *** *p* < 0.001) with STATISTICA 9.0 software (StatSoft, Tulsa, OK, USA).

## Data Availability

All data generated or analyzed during this study are included in this published article and its Supplementary Material.

## Supplementary Materials

The following supporting information can be downloaded at: https://www.mdpi.com/article/10.3390/ijms24021761/s1, Figure S1: Time-course assessment of mechanical allodynia, thermal hyperalgesia and locomotor activity after mild spinal cord injury (SCI); Figure S2: Reward-Seeking Behavior (RSB) test after mild spinal cord injury (SCI); Figure S3: Original scanned full blots for (A) IBA1 and (B) GFAP, and (C) respective actin expression in ACC shown in Figure 7; Figure S4: Original scanned full blots for (A) CX3CL1 and (B) CX3CR1, and (C) respective alpha-tubulin expression in ACC shown in Figure 8.
