# Supplementary material for: Transient Reflexive Pain Responses and Chronic Affective Nonreflexive Pain Responses Associated with Neuroinflammation Processes in Both Spinal and Supraspinal Structures in Spinal Cord-Injured Female Mice
Source: Int J Mol Sci. 2023 Jan 16;24(2):1761. doi: 10.3390/ijms24021761 (PMC9863935; doi:10.3390/ijms24021761)
Supplement: Supplementary file 1 [file ijms-24-01761-s001.zip › ijms-2128076-supplementary.pdf]

Supplementary Material

Transient Reflexive Pain Responses and Chronic Affective Nonreflexive Pain Responses Associated with Neuroinflammation Processes in Both Spinal and Supraspinal Structures in Spinal Cord-Injured Female Mice

Sílvia Castany [1,†], Anna Bagó-Mas [1,2,†], José Miguel Vela [3], Enrique Verdú [1], Karolina Bretová [2], Viktorie Svobodová [2], Petr Dubový [2] and Pere Boadas-Vaello [1,*]

[1] Research Group of Clinical Anatomy, Embryology and Neuroscience (NEOMA), Department of Medical Sciences, University of Girona, 17003 Girona, Catalonia, Spain
[2] Department of Anatomy, Division of Neuroanatomy, Faculty of Medicine, Masaryk University, 625 00 Brno, Czech Republic
[3] WeLab Barcelona, Parc Científic de Barcelona, 08028 Barcelona, Catalonia, Spain
* Correspondence: pere.boadas@udg.edu
† These authors contributed equally to this work.

Supplementary Material

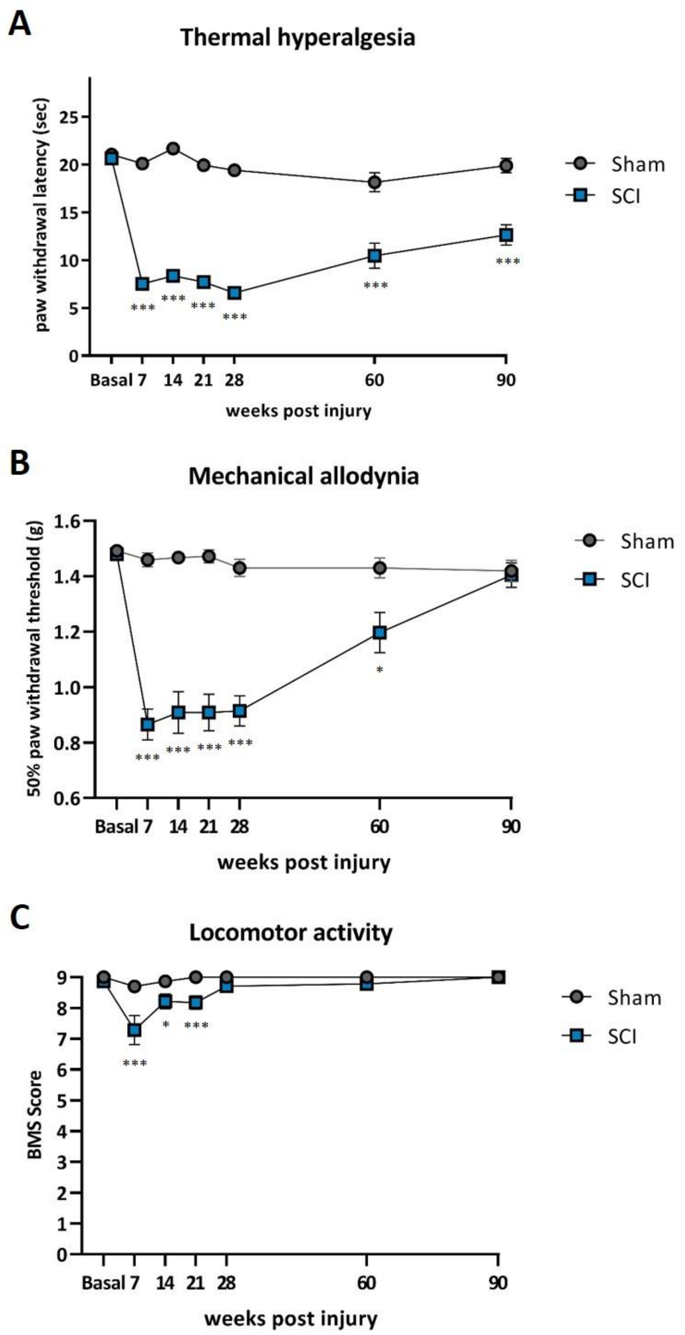

Figure S1. Time-course assessment of mechanical allodynia, thermal hyperalgesia and locomotor activity after mild spinal cord injury (SCI). Each point and vertical line represent the mean ± SEM. Experimental groups: Sham (n=15) and SCI (n=15). ** $p<0.01$ and ***$p<0.001$ by ANOVA test (Thermal hyperalgesia) or Mann-Whitney U test (Mechanical allodynia and Locomotor activity). (**A**) Thermal hyperalgesia was significantly evidenced in SCI animals up to 12 wpi. (**B**) Mechanical allodynia was significantly detected in SCI animals up to 8 wpi. (**C**) Mild BMS alterations referring to altered paw position but not to altered horizontal locomotion were detected in SCI at 1, 2 and 3 wpi. Afterwards, no further significant locomotor differences were detected between groups up to 12 wpi.

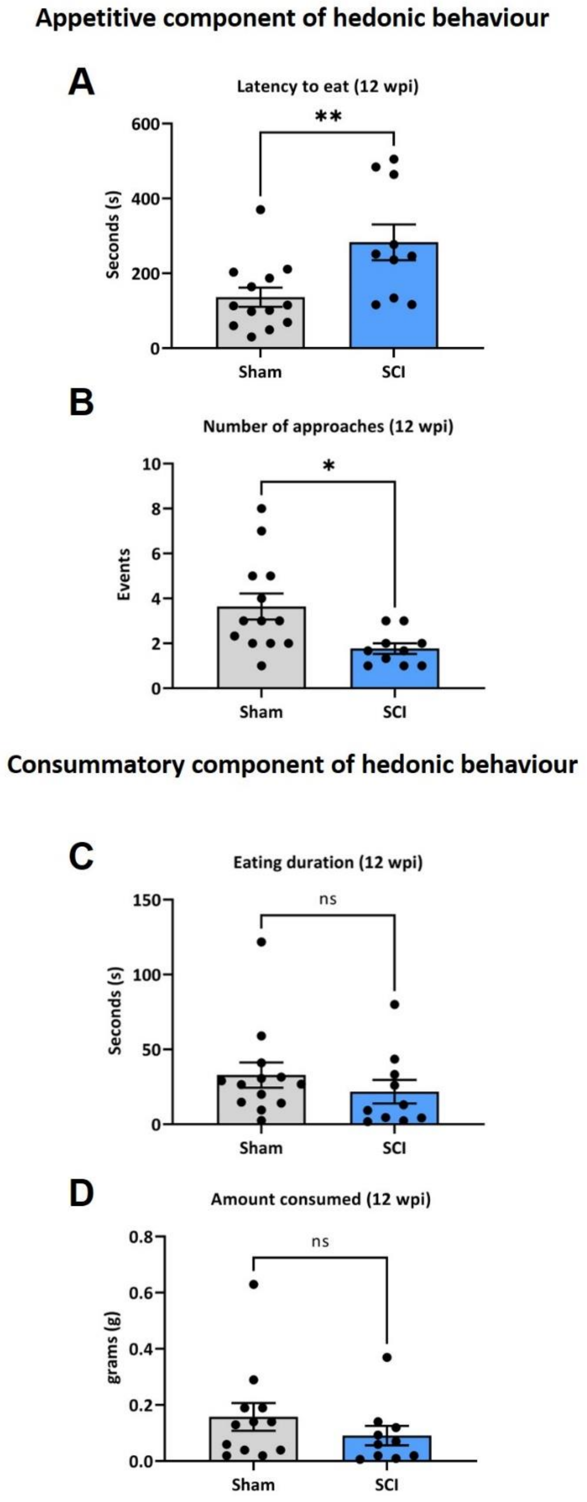

Figure S2. Reward-Seeking Behavior (RSB) test after mild spinal cord injury (SCI). Results are the mean ± SEM at 90 dpi. Experimental groups: Sham (n=13) and SCI (n=10). *p<0.05 and **p<0.01 by Unpaired t test. Motivational behavior disturbances were detected in SCI animals since significant differences in both (**A**) latency to eat and (**B**) number of approaches were detected. No significant differences were observed in (**C**,**D**) consummatory component.

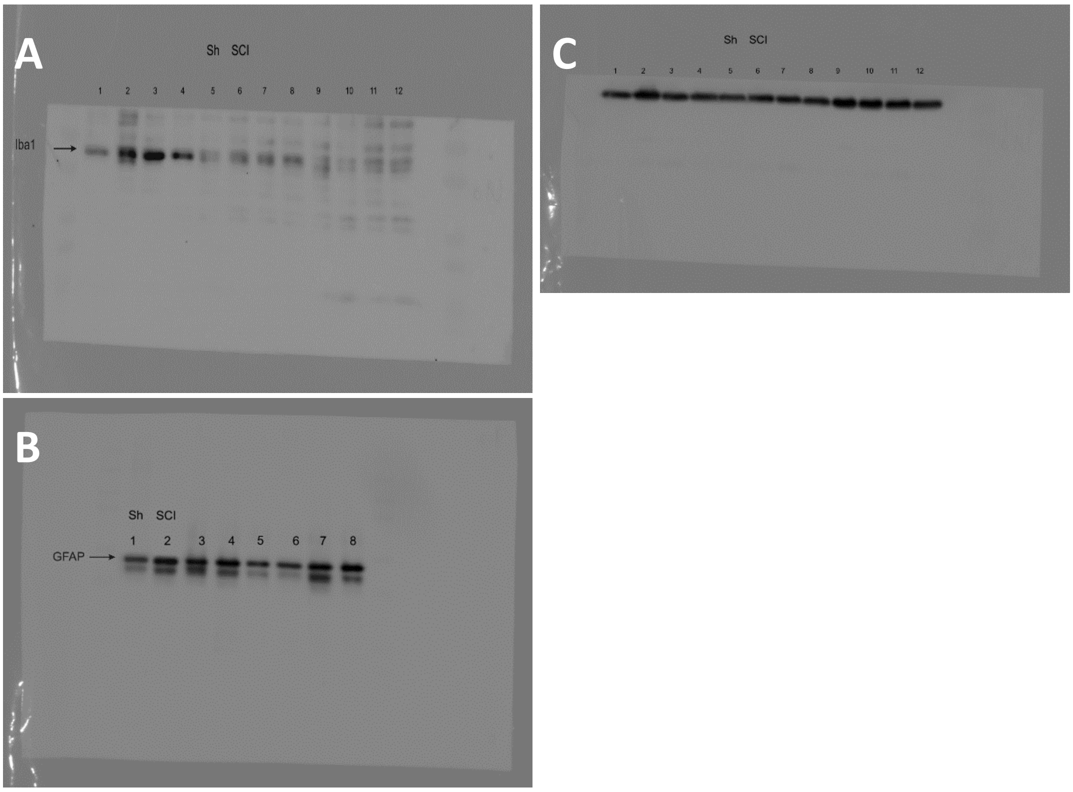

Figure S3. Original scanned full blots for (**A**) IBA1 and (**B**) GFAP, and (**C**) respective actin expression in ACC shown in Figure 7.

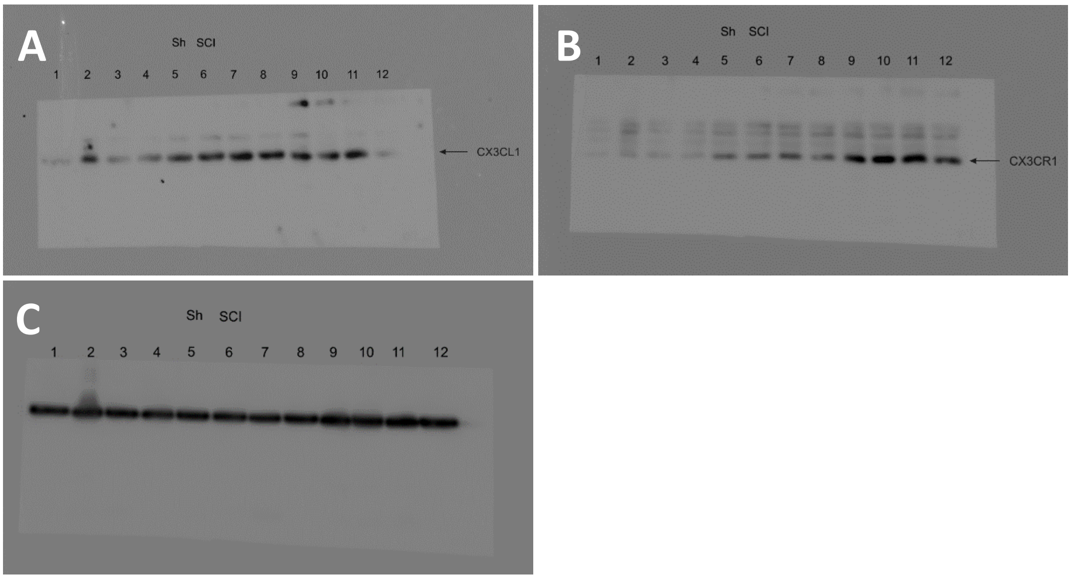

Figure S4. Original scanned full blots for (**A**) CX3CL1 and (**B**) CX3CR1, and (**C**) respective alpha-tubulin expression in ACC shown in Figure 8.
